# Toll-Like Receptor 4: A Promising Therapeutic Target for Alzheimer's Disease

**DOI:** 10.1155/2022/7924199

**Published:** 2022-08-21

**Authors:** Linyu Wu, Xiaohui Xian, Guangyu Xu, Zixuan Tan, Fang Dong, Min Zhang, Feng Zhang

**Affiliations:** ^1^Department of Rehabilitation Medicine, The Third Hospital of Hebei Medical University, Shijiazhuang 050051, China; ^2^Department of Pathophysiology, Hebei Medical University, Shijiazhuang 050051, China; ^3^Hebei Key Laboratory of Critical Disease Mechanism and Intervention, Shijiazhuang 050051, China; ^4^Department of Clinical Laboratory Medicine, The Third Hospital of Hebei Medical University, Shijiazhuang 050051, China

## Abstract

Alzheimer's disease (AD) is a progressive neurodegenerative disease that primarily manifests as memory deficits and cognitive impairment and has created health challenges for patients and society. In AD, amyloid *β*-protein (A*β*) induces Toll-like receptor 4 (TLR4) activation in microglia. Activation of TLR4 induces downstream signaling pathways and promotes the generation of proinflammatory cytokines, such as tumor necrosis factor-*α* (TNF-*α*), interleukin-6 (IL-6), and interleukin-1*β* (IL-1*β*), which also trigger the activation of astrocytes and influence amyloid-dependent neuronal death. Therefore, TLR4 may be an important molecular target for treating AD by regulating neuroinflammation. Moreover, TLR4 regulates apoptosis, autophagy, and gut microbiota and is closely related to AD. This article reviews the role of TLR4 in the pathogenesis of AD and a range of potential therapies targeting TLR4 for AD. Elucidating the regulatory mechanism of TLR4 in AD may provide valuable clues for developing new therapeutic strategies for AD.

## 1. Introduction

Dementia is the major cause of disability and death in people over 65 worldwide [1]. Approximately 50 million people worldwide have dementia, and this number will surpass 131 million by 2050 [2]. Alzheimer's disease (AD) is a complex age-related neurodegenerative disease and a major cause of dementia [3]. AD is characterized by progressive memory loss and learning deficits and is usually accompanied by language dysfunction, personality and behavioral changes, such as emotional apathetic state, depression and anxiety [4–7]. The AD incidence rate will markedly increase in the elderly with population ageing. Epidemiological studies have shown that the incidence of AD is about 10% in people over 65, rising to almost one-half in people over 85 [8]. This trend poses enormous challenges to AD patients and creates a heavy social burden [9].

AD is classified into two main forms: sporadic form and familial form. Nearly 90% of AD patients are sporadic. The condition impacts people at any age, but most individuals are over 65 years, often categorized as late-onset AD (LOAD) [10]. However, the cause of sporadic AD is still unclear. Familial AD usually occurs early and has a hereditary character. Genes encoding A*β* precursor protein (APP), presenilin 1 (PSEN1), and PSEN2 are causative genes [11]. The exact pathogenesis of AD is still unclear. Risk factors are aging, family history, unhealthy lifestyle, nutrition deficiency, and diabetes [12–14]. Among them, aging is the most well-known hazardous factor for AD [15]. Aging-associated cognitive dysfunction has become a predictable health threat in the elderly population. With society aging, AD could result in serious public health issues in the future.

The main pathological features of AD consist of amyloid plaques formed by abnormal aggregation of A*β* in the brain and neurofibrillary tangles (NFTs) composed of hyperphosphorylated tau protein in neuronal cells [16, 17], which is associated with a range of neurodegenerative events involving microglia cell activation, neurite dystrophy, neuroinflammation, oxidative injury, and mitochondrial disorder [17–19]. Although several studies have focused on AD therapy, clinical researches on treatments targeting A*β* or tau aggregation have produced unsatisfactory results [20]. The approved agents for AD treatment generally alleviate symptoms rather than address the underlying cause or pathogenesis [21]. Thus, it is urgent to investigate the molecular mechanisms and discover prevention and treatment strategies.

Toll-like receptors (TLRs) are transmembrane pattern-recognition receptors (PRRs) of the innate immune system that identify pathogen-associated molecular patterns (PAMPs), such as lipopolysaccharides (LPS), and damage-associated molecular patterns (DAMPs), such as high-mobility family protein box 1 (HMGB1) and A*β* [3, 22]. Stimulation of TLRs by injury factors results in severe inflammation via the release of proinflammatory cytokines, including IL-1*β* and interleukin-18 (IL-18) [23]. So far, studies have confirmed 10 functional TLRs (TLR1-10) in humans and 12 in mice (TLR1–9, 11–13) [24]. TLRs 1-9 can be expressed in human microglia [25]. Besides, TLRs are produced in various immune-associated cells, including monocytes, macrophages, microglia, and astrocytes, and nonimmune cells, such as endothelial and epithelial cells, in the brain parenchyma [26, 27]. TLR activation by pathogens and damaged cells triggers the phagocytic property of phagocytes/microglia to remove pathogens, injured tissues, and accumulated wastes [28–31]. TLR4 is the first TLR discovered in humans and the most studied member of the TLR family [32, 33]. As a neuroinflammatory receptor, TLR4 is expressed on astrocytes, microglia, and neurons in the brain and plays a key role in neuroinflammation in AD progression by recognizing exogenous and endogenous ligands [34, 35]. A*β*, a well-known exogenous ligand of TLR4, is reported to bind to TLR4 on the surface of microglia and astrocytes to trigger the release of proinflammatory factors [36, 37]. In AD mouse models, activation of TLR4 can enhance the clearance of amyloid plaques through phagocytosis of glial cells, thereby decreasing amyloid plaque load [38, 39]. In summary, the TLR4 signaling pathway plays an important role in the pathological mechanisms of AD. This review summarizes the relationship between TLR4 and AD to provide effective therapeutic targets for future research of AD.

## 2. Toll-Like Receptor 4 Signaling Pathway

**Figure 1:**
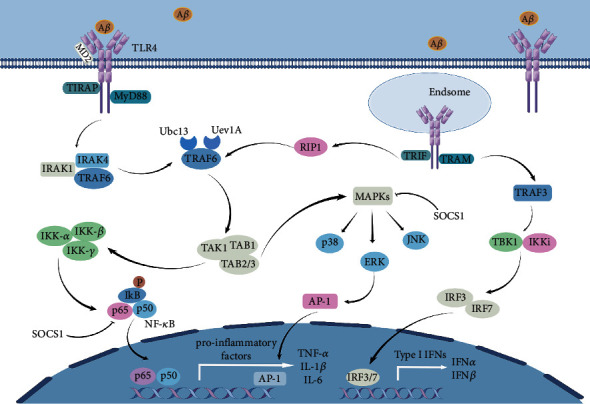
TLR4 binds to ligands and activates downstream pathways in both MyD88-dependent and MyD88-independent pathways. TLR4-MD2 recruits TIRAP and MyD88 and then signals to IRAKs. Then recruit TRAF-6, which with Ubc13 and Uev1A initiate the complex consisting of TAK1, TAB1, and TAB2/3 activation. The complexes of IKK-*α*, IKK-*β*, and IKK-*γ* are activated, promoting NF-*κ*B entry into the nucleus and leading to the release of proinflammatory factors, such as TNF-*α*, IL-1*β*, and IL-6. In addition, MAPKs are activated, and MAPKs-induced p38, ERK and JNK lead to AP-1 nuclear translocation. SOCS1 inhibits the TLR4 signaling pathway by affecting NF-*κ*B, MAPK activity, and p65 phosphorylation. TLR4-MD2 leads to endosome formation, resulting in TRAM translocation into the cytoplasm and activation of the TRIF-dependent signaling pathway. TRIF activates TRAF3 and the TBK1/IKKi complex, leading to phosphorylation of the interferon regulatory factors IRF3 and IRF7, which induce type I IFN gene expressions, such as IFN*α* and IFN*β*. Besides, TRIF interacts with RIP1, which activates the TAK1 complex and NF-*κ*B (this figure is made using the Figdraw).

As is shown in Figure 1, TLR4 is activated mainly through two pathways. One is dependent on the myeloid differentiation factor 88 (MyD88) signaling pathway, and the other is dependent on the Toll/interleukin-1 receptor- (TIR-) domain-containing adaptor inducing interferon-*β* (TRIF) signaling pathway. MyD88 is a common connector molecule that activates the inflammatory response. The MyD88-dependent signaling pathway is an essential stimulator of NF-*κ*B, which affects the subsequent expression of NF-*κ*B [40]. Before ligand-induced signaling occurs, TLR4 first needs to associate with its extracellular binding partner, myeloid differentiation factor 2 (MD-2) [41, 42]. The TLR4-MD-2 complex, on binding to the ligand, may recruit another TLR4-MD-2 pair and create a homodimeric state [40]. Then, the receptor multimer transmits intracellular signals by the TIR structural domain of TLR4 [43, 44]. TLR4-MD2 dimerization recruits TIR domain-containing adaptor protein (TIRAP) and MyD88. Signals from MyD88 are transmitted to the interleukin-1 receptor-associated kinase (IRAK) family of protein kinases via the interaction between MyD88 and the IRAK4 death domain [45] to recruit the tumor necrosis factor receptor-associated factor 6 (TRAF-6)[46–48]. Together with ubiquitin-conjugating enzyme 13 (Ubc13) and ubiquitin-conjugating enzyme E2 variant 1 (Uev1A), TRAF6 triggers the activation of a complex composed of transforming growth factor-*β*-activated kinase (TAK1), TAK1-binding protein 1 (TAB1), and TAB2, causing the phosphorylation of TAK1 and TAB2/3 [49, 50]. One part of the TAK1 pathway is the activation of the I*κ*B kinase (IKK) complex, which is an inhibitor of NF-*κ*B, including IKK-*α*, IKK-*β*, and IKK-*γ*. NF-*κ*B is composed of p65 and p50 dimers and is inactive if present in the cytoplasm with I*κ*B [51]. NF-*κ*B is activated, and proinflammatory factors (TNF-*α* and IL-1*β*) are released. The other part of the TAK1 pathway is the activation of mitogen-activated protein kinases (MAPKs), which involves p38, ERK, and JNK. MAPKs induce nuclear translocation of transcription factor complex AP-1, leading to the expression of cytokine genes [23, 52–54]. Moreover, the suppressor of cytokine signaling (SOCS1) is a cytokine-induced protein that negatively modulates cytokine signaling and directly downregulates TLRs signaling [55, 56]. SOCS1 can suppress TLR signaling by affecting NF-*κ*B, MAPK activity, and p65 phosphorylation. The TLR4 pathway through MyD88 is ‘early stage' NF-*κ*B activation, while TRIF-dependent activation is designated as ‘late stage' NF-*κ*B activation. The coordination of ‘early' and ‘late' signaling is specific to TLR4. The complex composed of TLR4-LPS causes the formation of the endosome, which leads to the translocation of TRAM into the cytoplasm and activation of TRIF-dependent signaling pathways [23]. The N terminus of TRIF activates the TNF-receptor-related factor 3 (TRAF3) and TANK-binding kinase/I*κ*B kinase (TBK1/IKKi) complex, resulting in phosphorylation of interferon regulatory factor 3 (IRF3) and IRF7, which triggers type I IFN gene expression, such as IFN*β*. There is crosstalk between TRIF-dependent and MyD88-dependent pathways [57]. TRAF6 is another downstream activation target of TRIF. The C-terminus of TRIF interacts with receptor-interacting serine/threonine-protein kinase 1 (RIP1). TRAF6 is activated by RIP1, which activates the TAK1 complex and NF-*κ*B [50, 57, 58]. In conclusion, TLR4 can cooperatively activate NF-*κ*B through TRIF-dependent and MyD88-dependent pathways, but type I IFN can only be generated by activating TRIF-dependent pathways [59, 60].

## 3. Evidence for the Involvement of TLR4 in AD

Emerging studies have reported that TLR4 plays a crucial role in the pathogenesis of AD. Genetic profiling of the human brain after death showed the upregulated TLR4 expression in the frontal cortex of AD compared with age-matched controls [61]. Moreover, TLR4 agonist, LPS, was identified in hippocampal lysates and neocortex of AD patients. The levels were two to three times higher than in age-matched control groups. In particular, LPS control levels were even 26 times higher in some cases of advanced AD [22]. These findings indicate that LPS from the microbiome or bacterial infections may accumulate in the brain, leading to AD. APP/PS1 double transgenic mice mimic progressive cognitive deficits and neuropathological changes in humans, which are dependable, easy to operate, and commonly used in AD studies. Higher p-Tau levels and A*β* aggregation in APP/PS1 mouse brains were related to increased IL-1*β*, IL-6, TNF-*α*, NF-*κ*B, and TLR4[62]. Likewise, TLR4 levels and IL-1*β* gene expression were significantly increased in hippocampal differentiated mice models without amyloidosis (i.e., entorhinal cortex damaged mice) compared with pseudodamaged mice [61]. The A*β*1-42 injection model is useful specifically in AD to explore the effect of inflammation [63]. Lateral ventricular injection of A*β* triggered inflammation, resulting in neuronal death, synaptic loss and cognitive dysfunction in WT mice but not in TLR4 knockout mice. Moreover, a selective TLR4 receptor antagonist inhibited A*β*-oligomer-induced microglial activation and memory dysfunction, which was not present in TLR4-deficient mice [64]. Therefore, the role of TLR4 in AD has attracted extensive attention.

TLR4 is closely associated with the occurrence of AD. Genetic association researches of TLR4 suggested that single nucleotide polymorphisms (SNPs) of TLR4 were associated with susceptibility to AD [65]. Asp299Gly polymorphism in the TLR4 gene could reduce inflammatory responses and prevent the development of sporadic AD. In the Italian cohort, a coding variant of TLR4 (rs4986790) was demonstrated to extend lifespan and reduce the risk of AD. Preclinical-stage familial AD (FAD) cases showed that the TLR4 variant was associated with a reduced risk of AD, better visuospatial and structural skills, and stable levels of IL-1*β* in cerebrospinal fluid over time [66–68]. Some TLR4 SNPs, such as rs10759930, rs12377632, rs7037117, and rs7045953, had neuroprotective effects in the Chinese Han population. rs11367 and rs1927907 were associated with an increased risk of AD [3]. Nevertheless, the exact function in AD of most of these gene variants has not been determined and still requires further research.

## 4. The Regulatory Mechanisms of TLR4 in AD

**Figure 2:**
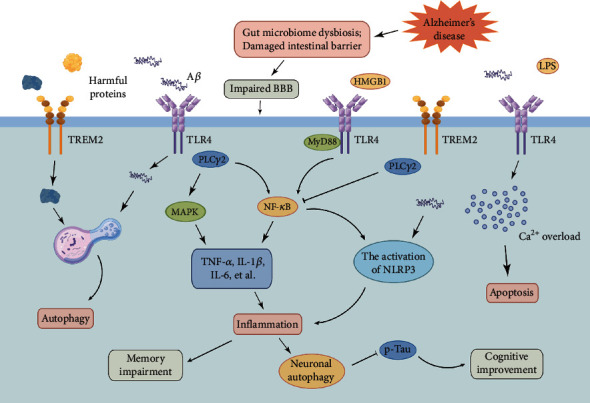
TLR4 mediates AD pathology by regulating inflammation, apoptosis, autophagy, and gut microbiota (this figure is made using the Figdraw).

As is shown in Figure 2, TLR4 regulates AD progression by modulating inflammation, apoptosis, autophagy, and gut microbiota homeostasis.

### 4.1. TLR4 Activation Contributes to Inflammation

From a physiological point of view, inflammatory reactions exert a protective effect on the brain, but excessive inflammation is detrimental. Neuroinflammation is closely related to the pathogenesis of AD [69]. The characteristic of neuroinflammation observed in AD is activated microglial cells around A*β* deposits [70]. Currently, microglia perform a double-edged role in the pathogenesis of AD. On the one hand, microglia may exert beneficial effects by removing A*β* plaques. They consume harmful extracellular proteins by TLRs and TREM2 and decompose them further by lysosomal-dependent means, including autophagy. Microglia promote the transformation of the ‘resting' type into an anti-inflammatory phenotype, including homeostasis, regeneration, and neuroprotection [71]. On the other hand, following the disruption of microglial phagocytosis and degradation, the persistence of A*β* plaques and the release of inflammatory factors convert microglia from an anti-inflammatory phenotype to a proinflammatory phenotype related to inflammation, neuronal injury, and death [72, 73]. Abnormal activation of microglia can liberate various proinflammatory or cytotoxic cytokines, such as TNF-*α*, IL-1*β*, IL-6, nitric oxide (NO), reactive oxygen species (ROS), cyclooxygenase-2 (COX-2), and prostaglandin E2 (PGE2), which can significantly accelerate neuroinflammatory and neurotoxic reactions. Neuroinflammation ultimately results in neuronal cell death, synaptic degeneration, and cognitive impairment [74–78]. Currently, targeting neuroinflammation has become one of the essential therapeutic goals for AD, and understanding the mechanism underlying neuroinflammation in AD is a crucial breakthrough point for exploring the pathogenesis of AD. Researches have shown that TLR4 plays a key role in the pathogenesis of inflammation. The regulation mechanism of TLR4 on inflammation in AD is described below.

#### 4.1.1. The Relationship Between A*β* and TLR4 in AD

In AD, insoluble A*β* macrofibrils and oligomers can be recognized by the innate immune system in the brain as a dangerous signal, resulting in activation of the innate immune response [79]. A*β*, the main toxic protein in AD patients, can trigger microglial activation and release of inflammatory cytokines in the brain, finally leading to AD-related neuroinflammation [80]. Studies have shown that A*β* combines with TLR4 on the surface of microglia and astrocytes and then activates the NF-*κ*B and MAPK signaling pathways and triggers the release of proinflammatory factors, such as TNF-*α*, IL-1*β*, and IL-6 [81–83]. Overexpression of TLR4 was triggered by stimulation of aging rat neurons with A*β* oligomer, which made cells sensitive to subsequent TLR4 stimulation. In vitro, supernatant from A*β*-stimulated microglial cells was added to primary murine neurons, and TLR4 was found to be involved in A*β*-regulated microglia neurotoxicity and upregulating TNF-*α*, IL-1*β*, IL-10, and interleukin-17 (IL-17)[84]. In addition, LPS-cultured microglia incubated with A*β*42 for 24 h reduced A*β*42 in the culture medium by 50%, suggesting that TLR4 increased A*β* clearance [38]. This finding also implies that microglia can be activated through the TLR4 pathway to suppress A*β* deposition in the early stages of *β*-amyloidosis, thus protecting neurons against A*β*-mediated neurotoxicity [85]. Following the disease progression continued exposure of microglia to A*β* mitigates TLR4 response and activated microglia cannot clear A*β* deposition [86]. In cocultured neuron-glia cells, A*β* oligomers- (A*β*o-) driven neuronal cell death was primarily attributed to the A*β*-sensitized TLR4 pathway of astrocytes and microglia through autocrine/paracrine mechanisms, suggesting another mechanism through TLR4-mediated A*β* toxicity. Studies showed that TLR4 was involved in A*β*o-mediated memory loss in AD. A single lateral ventricle injection of A*β*o in C57BL/6J mice activated glial cells, resulting in increased expression of pro-inflammatory factors and significantly impaired recognition and memory. TLR4 receptor antagonists eliminated the harmful effects of A*β*o on memory, and A*β*o had no effect on glial cell activation or memory in TLR4-knockout mice [64]. In summary, A*β* is involved in the progression of inflammation in AD through the TLR4 signaling pathway and may impact memory.

#### 4.1.2. The Relationship Between HMGB1 and TLR4 in AD

High-mobility family protein box 1 (HMGB1) is a typical DAMP released by necrotic or excitatory neurons. HMGB1 protein is involved in initiating and activating neuroinflammation under pathologic conditions and the pathogenesis of neurodegenerative disorders, such as AD. Research suggested that the expression of HMGB1, RAGE, TLR4-NF-*κ*B, and inflammatory cytokines increased in the nucleus and cytoplasm of hippocampal neurons in A*β*25-35-induced AD-associated neuroinflammation models [87]. HMGB1 exerted its biological property by directly combining with receptors for advanced glycation end products (RAGE) and TLR4, acting as a chemoattractant or proinflammatory factor [88]. HMGB1 gene silencing alleviated inflammation induced by A*β* in hippocampal neuron cultures [87]. Extracellular HMGB1 acted as a chaperone of A*β*, decreased microglial A*β* clearance, and interacted with RAGE and TLR4, which participated in microglial A*β* phagocytosis, leading to AD progression [38, 89, 90]. Notably, subcutaneous injections of anti-HMGB1 antibodies prevented neurodegeneration and reversed cognitive deficits, even in the presence of A*β* plaques [91].

Disruption of learning and memory are important features of AD. In the new object recognition test (NORT), a decrease in the new object preference index demonstrated the effect of HMGB1 injection on memory encoding. When HMGB1 was injected into control mice and TLR4 and RAGE knockout mice, the damage to memory coding was similar [92]. In addition, TLR4 antagonists blocked the amnesic role of HMGB1 in RAGE knockout mice [92]. These studies suggest that HMGB1-induced memory dysfunction is regulated by RAGE and TLR4, but the exact mechanisms are still unclear. Myristoylated alanine-rich C-kinase substrate (MARCKS), a submembrane protein, helps stabilize the actin network. Studies have shown that HMGB1 released by necrotic or overexcited neurons binds to TLR4 and activates MAPKs, inducing MARCKS phosphorylation to initiate neurite degeneration, resulting in impaired memory function [91]. Thus, these results suggest that HMGB1 is involved in AD progression through the TLR4 pathway.

#### 4.1.3. The Relationship Between TREM2 and TLR4 in AD

TREM2 is a transmembrane receptor (one of the members of the TREM family) expressed on the surface of several myeloid cells, including microglia, monocytes, and macrophages. It is an important innate immune receptor in the brain, which regulates microglia survival, proliferation, biosynthesis, and factor release; moreover, it has a protective effect on A*β* pathology [93, 94]. Reports indicated that TREM2 was expressed at higher levels in the brains of AD patients than in normal controls [95]. Polymorphisms in TREM2 genes were associated with a higher risk of LOAD [96]. Recent epidemiological surveys suggested that serum levels of TREM2 might serve as a prospective new predictive biomarker for the incidence of dementia [97, 98]. Studies have shown that TREM2 plays an important role in the protective mechanisms of AD, in which TLRs play a key role. Reports showed that TREM2 negatively regulated TLR4-mediated inflammation [99, 100]. The APP/PS1 mice had higher levels of TLR4 and TREM2 in their brains; TLR4 showed continuous upregulation, and TREM2 levels significantly decreased in APP/PS1 mice after LPS treatment, suggesting that LPS-induced TLR4 hyperactivity inhibited the negative regulation of inflammation in TREM2[100]. Moreover, overexpression of TREM2 in microglia inhibited TLR4 levels, leading to altered expression of downstream effectors of TLR4 (ERK, p38, and p65) and proinflammatory factors (IL-6, IL-1*β*, and TNF-*α*), whereas silenced TREM2 gene elevated TLR4 levels [101]. These results suggest that TREM2 inhibits neuroinflammatory responses by downregulating the TLR4 pathway.

Phospholipase C*γ*2 (PLC*γ*2) is an intracellular enzyme that can cleave membrane phosphatidylinositol-4,5-diphosphate (PIP2). The variation of the PLC*γ*2 gene is associated with AD [102]. Recent studies have shown that PLC*γ*2 regulates various microglial functions via numerous upstream molecules, such as TREM2 and TLR ligands. TREM2 alleviated PLC*γ*2-mediated inflammation, and TLR4/PLC*γ*2-reliant inflammatory signals were amplified without TREM2 [2]. The exact relationship between TREM2 and TLR4 in AD needs further study.

#### 4.1.4. The Relationship Between NLRP3 and TLR4 in AD

The activation of nod-like receptor protein 3 (NLRP3) inflammasome is an early pathogenic AD event. Extracellular fiber A*β* activated the typical inflammasome pathway by triggering the TLR4/MyD88/NF-*κ*B signal pathway. Besides, oligomers and fiber A*β* could interact directly with NLRP3 and ASC, leading to activation of NLRP3 inflammasome [103]. Microglia activation was induced by injections of fibrin A*β* into the striatum of mice, whereas microglia activation was inhibited in MyD88-deficient or ASC-deficient mice, indicating that aggregated A*β* produced a signaling cascade, including MyD88 and NLRP3 inflammasome [104]. Furthermore, MyD88 deletion reduced microglial activation and brain A*β* load and improved behavioral dysfunction in APP/PS1 mice [105, 106]. These findings indicate that the TLR4/MyD88 pathway participates in the initiation of NLRP3 activation in AD mouse models.

### 4.2. TLR4 Activation Contributes to Neuronal Apoptosis in AD

A*β*o facilitates Ca2+ entry and mitochondrial Ca2+ overload, resulting in neuronal cell death. As a TLR4 receptor agonist, LPS enhanced the concentration of cytoplasmic Ca2+, resulting in apoptosis. Studies have found that LPS or A*β*o exposure for 48 h could not lead to apoptosis of young cultured cells. The combination of A*β*o and LPS enhanced Ca2+ response and neuronal death in cultured young rat neurons, and this effect significantly increased in aged neurons [107]. These data suggested that Ca2+ signals activated by TLR4 and induced by A*β*o might crosstalk with each other, thereby boosting cell death in the neurons, especially in senescence. This finding may be related to A*β*o-induced changes in TLR4 expression. In conclusion, the TLR4 signaling pathway is closely associated with AD by triggering apoptosis.

### 4.3. TLR4 Activation Contributes to Neuronal Autophagy in AD

Autophagy is a mechanism that promotes the clearance of damaged organelles or abnormal intracellular protein aggregation related to multiple CNS diseases involving tau and A*β* in AD. Transcription of autophagy-associated genes, such as Beclin1, ATG5 and ATG7, was downregulated in the elderly brain [108]. Studies demonstrated that promoting autophagy could prolong the longevity of numerous organisms, from yeast to mice, and attenuate the progression of age-related disorders. Therefore, autophagy may provide protective roles, especially on aging neurons. There is increasing evidence of impaired autophagy in AD [109–111]. Histological analysis revealed the accumulation of autophagosomes and autolysosomes in AD brain neurons [112]. Studies showed that TLR4 ligand promoted or suppressed autophagy, which might had opposite roles on protein and organelle turnover. Qin et al. found that chronic mild TLR4 stimulation might boost neuronal autophagy, reduce brain p-tau protein levels, and ameliorate cognitive dysfunction in transgenic AD mice [39]. Contrarily, reports showed that chronic TLR4 activation activated premature organelle autophagy, leading to impaired neuronal signal and cell death [113]. Moreover, sustained IL-4-induced activation of TREM2 led to increased lysosomal and LC3II/I expression, increased microglial autophagy, and impaired CARD9-TLR4 pathway, contributing to improved cognitive function in AD mice. Thus, the TLR4 signaling pathway is closely related to AD by regulating autophagy.

### 4.4. TLR4 Activation Regulates Gut microbiota in AD

About 37 trillion microbes reside in the human body; 70% of these microbes are found in the gut [114]. These microorganisms can protect the host from pathogen invasion, facilitate digestion and absorption of the host, and modulate drug metabolism [115]. Recent research has shown that histological and behavioural presentations of AD are associated with dysbiosis of the gut microbiome. In an AD mouse model, antibiotic-induced perturbations of gut microbial diversity affected neuroinflammation and amyloidosis. Changes in gut microbiome composition due to aging may result in chronic low-grade inflammation, a hazardous factor associated with cognitive decline in older adults. Brain amyloidosis in cognitively impaired older adults was associated with proinflammatory intestinal flora and markers of peripheral inflammation. AD was associated with damage to the intestinal barrier, through which LPS and proinflammatory factors produced by pathogens entered the body, disrupted the integrity of the blood-brain barrier (BBB), enhanced the uptake of A*β* and *α*-syn in the brain, and activated microglia-induced immune reactions through the LPS/TLR4/NF-*κ*B pathway, resulting in neuronal loss [116, 117]. Studies have suggested that the effects of aging gut microbiome dysregulation on cognitive functioning may be related to the LPS-activated TLR4/NF-*κ*B signaling and neuroinflammation in the brain. These findings suggest that inflammation, apoptosis, autophagy, and gut microbiota are closely related to AD via triggering TLR4 signaling. Therefore, a better understanding of the regulatory mechanism of TLR4 in AD may help identify potential therapeutic approaches to prevent AD.

## 5. Application of Therapies Targeting TLR4 in AD

As represented in Table 1, studies showed that multiple AD therapies targeting TLR4 exerted protective effects primarily by inhibiting the expression of TLR4 signaling pathway molecules, suppressing microglia activation, reducing neuronal death, and improving learning and memory function.

### 5.1. Flavonoids

Flavonoids are most helpful for treating neurodegenerative diseases due to their anti-inflammatory, antioxidant, and antiapoptotic properties [118, 119]. Sinensetin (SIN), a polymethoxyflavonoid, is a significant active compound predominant in citrus plants and indicated to have antitumor, antioxidant, and anti-inflammatory pharmacological activities [120–123]. Zhi et al. found that SIN preconditioning inhibited A*β*25-35-regulated upregulation of TLR4 and nuclear translocation of NF-*κ*B p65. SIN protected SH-SY5Y cells from A*β*25-35-regulated neurotoxicity by inhibiting oxidative stress, inflammation, and apoptosis via suppressing the TLR4/NF-*κ*B pathway [124]. Moreover, overexpression of TLR4 eliminated the protective role of SIN. Hesperidin is a bioactive flavonoid produced by hesperidin and found in citrus fruits [125]. Reports showed that hesperidin exerted neuroprotective effects in diverse models [126, 127]. Hesperidin is a powerful free-radical scavenger that facilitates cellular anti-inflammatory properties [128]. Muhammad et al. found that hesperidin significantly inhibited the expression of TLR4, P-NF-*κ*B, TNF-*α*, and IL-1*β* induced by A*β*1-42, showing that hesperidin had the same effect as specific inhibitors [129]. This finding demonstrated that hesperidin effectively mitigated A*β*-induced pathological changes in mice and cells by modulating TLR4/NF-*κ*B to inhibit neuroinflammation and was a promising and reasonable neuroprotective agent. Soybean isoflavone (SIF) is a type of soybean phytochemical with anti-inflammatory, antioxidant, and antiosteoporosis properties. Youn et al. demonstrated that plant polyphenols could suppress LPS-induced TLR4 dimerization and thus regulate TLR-regulated inflammation [130]. As a polyphenol, SIF may also act on TLR4 and suppress the toxicity of A*β*, further attenuating downstream inflammatory pathways. Ding et al. demonstrated that SIF treatment significantly reversed A*β*1-42-induced elevated expression of TLR4 and NF-*κ*B p65 subunit, decreased expression of TNF-*α* and IL-1*β*, improved A*β*-induced brain injury, and ameliorated learning and memory in rats [131]. These results suggested that SIF could play a neuroprotective effect in AD through the TLR4/NF-*κ*B signaling pathway, which was beneficial to AD treatment. In summary, these flavonoids can inhibit the expression of TLR4 and play a significant role in AD.

### 5.2. Medications

#### 5.2.1. Circumdatin D

Acetylcholine (ACh) is a crucial neurotransmitter in memory and learning processes. The functional decline of cognition in AD patients is related to a lack of the neurotransmitter Ach in the brain. Acetylcholinesterase (AChE) is a hydrolytic enzyme that hydrolyses acetylcholine to choline and acetic acid [132, 133]. The study found a 20% increase in plasma levels of AChE in AD patients compared to age- and sex-matched controls [134]. Suppression of AChE averted the breakdown of ACh and subsequently added its concentration and duration of action, thought to be of clinical benefit for patients with AD. AChE inhibitors currently account for four of the five treatments prescribed for AD. These cholinergic drugs are less effective in curing the disease and can only relieve some AD symptoms by facilitating cholinergic signaling [135, 136]. Therefore, it is essential to develop novel acetylcholinesterase inhibitors for therapeutic intervention in AD. Natural products (particularly alkaloids) are potential sources for novel AChE inhibitors [137]. Circumdatins are a group of alkaloids of marine origin with dual inhibitory activities of AChE and proinflammatory reactions. Among them, circumdatin D showed a promising neuroprotective effect through the multitarget strategy. Zhang et al. found that cyclostatin D regulated TLR4-mediated NF-*κ*B, MAPKs, and JAK/STAT signaling pathways associated with inflammation in LPS-stimulated BV-2 cells and protected primary neurons against LPS-regulated neurotoxicity [138]. Therefore, circumdatin D may be a potential drug to exert the neuroprotective effect of AD via TLR4.

#### 5.2.2. GX-50

Sichuan pepper is one of the main spices used in Chinese food and has anti-inflammatory, antifungal, and analgesic activities [139]. GX-50, a natural ingredient extracted from Sichuan pepper, is a promising agent for AD therapy [140–142]. The overactivated microglial cells, proinflammatory factors, and continued deposition of A*β* plaques led to a positive feedback loop of persistent and irreversible inflammation, ultimately leading to the progression of AD. Studies have suggested that GX-50 may be involved in this inflammatory circuit. Studies showed that GX-50 could directly decrease the aggregation of A*β* oligomer, reduce neuroinflammation, and improve the cognitive function of APP transgenic mice. Shi et al. found that GX-50 exerted an anti-inflammatory role on A*β*-induced microglial overactivation through a mechanism involving the TLR4-mediated NF-*κ*B/MAPK signaling pathway. GX-50 inhibited A*β*-stimulated activation of TLR4, subsequently inhibited MyD88 and TRAF6 recruitment, resulting in inhibition of NF-*κ*B and MAPK, and inhibited A*β*-induced inflammatory reaction [73]. These results suggest that TLR4/NF-*κ*B/MAPK may be an important pathway in GX-50 treatment of AD.

#### 5.2.3. Resveratrol

Several epidemiological data suggest that red wine consumption in moderation is related to a lower incidence of dementia and AD [143–145]. Studies in mouse AD models have shown that red wine consumption reduced brain amyloid deposition and A*β*-related cognitive impairment [146, 147]. Resveratrol is a natural polyphenol found in red wine. Recent studies have shown that this polyphenol is related to antiamyloidosis and neuroprotective activities of cell lines in vitro and mice in vivo. The relative abundance of resveratrol found in red wine is related to possible neuroprotective effects, thus interpreting the advantageous roles of wine consumption on AD. Resveratrol may have anti-inflammatory activities in multiple systems, involving activated microglia [148]. Evidence showed that resveratrol significantly reduced A*β*-induced microglial inflammation in vitro and in vivo. Resveratrol effectively inhibited LPS-regulated inflammation in microglia BV-2 cells and RAW 264.7 macrophages. Resveratrol preferentially antagonized the IKK/I*κ*B*α*/NF-*κ*B signaling pathway under LPS stimulation, repressed NF-*κ*B transcription and the expression of multiple NF-*κ*B target genes, such as TNF-*α* and IL-6. Moreover, oral resveratrol reduced the number of activated microglia related to cortical amyloid plaque formation in an in vivo study of a mouse model of amyloid deposition in the brain [149]. In conclusion, resveratrol negatively controls A*β*-induced microglial inflammation in vitro and in vivo, which may be associated with TLR4-related signaling pathways and is a potential treatment for AD.

### 5.3. Bacteria Associated with the Gut

Probiotics offer a potential preventive effect on AD progression [150, 151]. Multiple probiotics reduce the Mini-Mental State Examination (MMSE) score and some metabolic characteristics in patients with AD. A probiotic mixture was identified to regulate the gut microbiome and ameliorate memory dysfunction and oxidative stress in rats injected with *β*-amyloid protein (1e42)[152]. Probiotic-4 is a probiotic preparation made of *Lactobacillus casei*, *Bifidobacterium lactate*, *Lactobacillus acidophilus*, and *Bifidobacterium bifidum*. Studies have shown that probiotic-4 regulates age-related gut microbiome dysregulation improving microbiome axis defects and cognitive dysfunction. The mechanism of probiotic-4 neuroprotective effects was related to inhibition of TLR4 and RIG-I mediated NF-*κ*B pathway and inflammatory reactions [153]. Glucagon-like peptide-1 (GLP-1) is a type of endogenous hormone secreted by endocrine cells in the ileum that facilitates insulin secretion and suppresses glucagon secretion, lowering blood glucose in a glucose-dependent manner [154]. Studies have shown that GLP-1 plays a neuroprotective role in affecting the proliferation and apoptosis of neural cells; ameliorating learning, memory, and motor deficits; reducing the deposition of A*β* plaques in the brain; reducing the loss of dopaminergic neurons; and promoting neural regeneration [155]. GLP-1 also reduced hyperphosphorylated tau and neurofilament proteins in rodents to improve AD-like neurodegeneration, which was related to memory amelioration and learning dysfunction [156]. GLP-1 has demonstrated efficacy in clinical trials in patients with AD [157, 158]; however, GLP-1 undergoes easy degradation, and therefore, continuous intravenous infusions or continuous subcutaneous injections are necessary to perform therapeutic roles, thus limiting its clinical application [154]. The engineered strain may be a novel intervention for treating AD through alleviating neuroinflammation. Fang et al. constructed an engineered *Lactococcus lactis strain* MG1363-PMG36E-GLP-1 capable of continuously expressing GLP-1. They found that MG1363-PMG36E -GLP-1 reduced neuroinflammation by downregulating the TLR4/NF-*κ*B signaling, upregulated the AKT/GSK3*β* pathway, and restored the disturbed microbiome to normality, remarkably alleviating spatial learning and memory impairment in AD mice [159]. These results provide theoretical support for applying GLP-1 engineered strain in treating AD through TLR4.

### 5.4. Molecular Inhibitors and Agonists

#### 5.4.1. TAK-242

TAK-242 is a selective inhibitor of TLR4 with a low molecular weight that destroys the interaction of TLR4 with the cohesive molecules and suppresses its downstream pathway. Due to its high lipid solubility and low molecular weight, TAK-242 has the ability to cross the BBB [160]. Cui et al. found that TAK-242 could protect APP/PS1 transgenic AD mice from injury. They proved that TLR4 expression increased in mice with AD, and inhibition of TLR4 triggered microglia from an inflammatory M1 phenotype to a protective M2 phenotype and protected neurons from cytotoxicity of activated BV2 microglia by downregulating MyD88/NF-*κ*B and NLRP3 signaling pathways in AD [33].

#### 5.4.2. Monophosphoryl Lipid A (MPL)

Monophosphoryl lipid A (MPL), a chemically detoxified part of the lipid A, is an LPS-derived TLR4 agonist that displays distinct immunoregulatory activities at nonpyrogenic doses [161, 162]. Moreover, MPL is secure in humans, and millions of patients have received it as part of several vaccine formulations, including Cervarix [163]. Studies have shown that chronic systemic administration of MPL can stimulate the phagocytosis of innate immune cells, induce a moderate inflammatory response, reduce the brain A*β* load, and improve the cognitive function of AD mice. These results have shown that MPL stimulates p38 and facilitates A*β* uptake selectively while preventing the production of potentially harmful proinflammatory factors triggered by other pathways [162]. Studies showed that p38, ERK, JNK, and NF-*κ*B induced TLR-mediated factor production, but p38 could upregulate scavenger receptor expression and induce phagocytic activity [164, 165]. Although MPL did not stimulate ERK or JNK and induced NF-*κ*B to a lower extent than LPS, it strongly activated p38 and drove SR-A expression in microglia. These findings showed that MPL stimulated p38, facilitated A*β* uptake selectively, and avoided the production of potentially harmful proinflammatory factors triggered by other signal pathways. TLR4 stimulates the detoxification ligand MPL to significantly mitigate AD-related pathology, and its application in treating AD remains to be explored. Taken together, molecular inhibitors and agonists focusing on the regulation of the TLR4 may be potential treatment strategies for AD.

### 5.5. Clinical Drugs

#### 5.5.1. Alogliptin

Type 2 diabetes mellitus (T2DM) is the main risk factor for progression to AD. Therefore, antidiabetic agents may be effective treatments for AD. Dipeptidyl peptidase-4 (DPP-4) suppressors are widely known antidiabetic agents for managing T2DM via attenuating endogenous GLP-1 degradation, which leads to inhibition of glucagon release and glucose-dependent rise in insulin secretion [166]. In addition to the glycemic effect, DPP-4 suppressors have neuroprotective effects. Among them, sitagliptin, vildagliptin, and saxagliptin inhibited the accumulation of A*β* and abnormal phosphorylation of tau to alleviate inflammation and reverse behavioral defects in AD rats [167, 168]. Studies showed that alolliptin, a highly selective and effectual DPP-4 suppressor, could ameliorate cognitive dysfunction and depressive symptoms in obese ApoE-/- mice. Ayman et al. found that alolliptin reversed the LPS-activated TLR4/MyD88/NF-*κ*B signaling pathway and reduced TNF-*α* and IL-6 levels, thus playing a neuroprotective role [169]. This finding suggests that alolliptin may improve amyloidation-related cognitive decline by inhibiting TLR4 activation-related neuroinflammation and may be a potential treatment for AD.

#### 5.5.2. Cattle Encephalon Glycoside and Ignotin (CEGI)

Cattle encephalon glycoside and ignotin (CEGI) involves monosialotetrahexosyl ganglioside (GM1), hypoxanthine, free amino acids, and polypeptides [170]. In China, CEGI injections are commonly used for treating central and peripheral nerve injury disorders, such as acute ischemic stroke and diabetic peripheral neuropathy [171, 172]. Gao et al. found that CEGI inhibited activated microglia in APP/PS1 mice, suggesting that CEGI may be a prospective agent for treating AD. In addition, they showed that CEGI inhibited elevated TLR4 levels in APP/PS1 mice and A*β*-induced BV2 cells and inhibited NF-*κ*B p65 phosphorylation in vitro and in vivo [173]. Based on these results, the authors indicated the role of CEGI in antineuroinflammation by inhibiting the TLR4/NF-*κ*B signaling pathway. This result provides new theoretical support for applying CEGI in managing AD.

#### 5.5.3. Fasudil

Several studies have shown that the Rho/Rho kinase (ROCK) signaling pathway participates in many pathological processes, such as oxidative stress, inflammatory cell migration, and immune cell activation, which may influence the occurrence and progression of various neurodegenerative diseases, such as AD. Studies showed that ROCK inhibitors could prevent neuronal injury, neuroinflammation, and demyelination. Thus, ROCK has become a possible target for new therapies for AD [174, 175]. Fasudil is an effective ROCK inhibitor shown to have several neuroprotective effects in the CNS: promoting axonal regeneration, inhibiting inflammatory responses, and protecting against A*β*-induced neurodegeneration [176–178]. Guo et al. found that microglia-astrocyte crosstalk through the TLR4/MyD88/NF-*κ*B signaling pathway played an important role in the neuroinflammation of AD. They observed that fasudil inhibited the expression of TLR4, MyD88, and NF-*κ*B in APP/PS1 Tg mice, thus suppressing microglia activation and inducing their transformation to an anti-inflammatory phenotype and further transforming astrocytes from an A1 phenotype to an A2 phenotype; the cognitive defects of APP/PS1 Tg mice showed improvement [179]. These results suggest that fasudil may play a therapeutic role in AD through the TLR4 signaling pathway.

#### 5.5.4. Atorvastatin

Statins are 3-hydroxy-3-methylglutaryl-coA (HMGCoA) reductase suppressors and the most efficacious cholesterol-lowering drugs of all lipid-lowering medicines [180]. Clinical data showed a decrease in the incidence of AD among statin users compared to nonusers [181]. Studies have shown that the main effect of statins on AD patients is to reduce the inflammatory reaction caused by microglial activation rather than the accumulation of A*β* [182]. Atorvastatin, a lipophilic member of the statin family, has shown a promising therapeutic anti-inflammatory role [183]. Research showed that after 3 weeks of atorvastatin pretreatment, the levels of the anti-inflammatory factor IL-4 enhanced in the hippocampus and the intracellular expression of A*β*-induced proinflammatory factors TNF-*α*, IL-6, and IL-1*β* decreased [184, 185]. We found that chronic treatment with high-dose atorvastatin significantly reduced the levels of TLR4, TRAF6, and NF-*κ*B, inhibited the activation of microglia and astrocytes, alleviated hippocampal pathologic alterations and neuronal apoptosis, and improved spatial learning ability and memory impairment in AD rats. They also found a tendency for continuous low-dose atorvastatin administration to ameliorate A*β*-induced cognitive dysfunction; however, the difference was not prominent, suggesting that the neuroprotective role of atorvastatin in AD appears to depend on the dose and treatment regimen used [37]. In conclusion, appropriate modification of the TLR4-mediated signal pathway may be one of the potent targets for atorvastatin treatment of A*β*-regulated neurotoxicity in AD.

### 5.6. Traditional Chinese Therapy

#### 5.6.1. Chinese Herbal Medicine

*(1) Tetrandrine*. Tetrandrine is a natural product isolated from the Chinese herbal medicine *Stephania tetrandra*, which has many biological activities [186]. Previous studies have demonstrated that tetrandrine has anti-inflammatory properties in various cell types, such as astrocytes, T cells, and monocytes.[187–190]. Yang et al. reported that tetrandrine inhibited inflammatory activation of LPS-induced microglia in culture [191]. He et al. demonstrated that tetrandrine ameliorates cognitive disorder by suppressing NF-*κ*B activity and inflammation in a rat model of AD induced by A*β*1-42 [192]. They found that in 5XFAD mice, tetrandrine administration dose-dependently reduced amyloid plaque deposition in the brain, diminished apoptosis in the hippocampus, and reduced cognitive performance. Tetrandrine repressed the expression of TLR4 and p65 in BV2 cells induced by A*β* 1-42 and effectively suppressed the inflammatory activation of BV2 cells [193]. These results suggests that tetrandrine may promote the clearance of amyloid plaques by regulating the functional transformation of microglia. Together, these findings indicate that tetrandrine may be a promising agent for treating AD through TLR4-mediated neuroinflammation.

*(2) Pinoresinol diglucoside (PDG)*. *Eucommia ulmoides*, a traditional Chinese herbal medicine, is widely used in China. Pinoresinol diglucoside (PDG), extracted form of *Eucommia ulmoides*, is one of the primary lignans with anti-inflammatory, antioxidant, antivascular injury, anticancer, antihypertensive actions, and prevention of osteoporosis [194–200]. Studies have reported that PDG inhibits NF-*κ*B and induces Nrf2-dependent HO-1 activation by regulating the MAPK, PI3K/Akt, and GSK-3*β* pathways to suppress the proinflammatory reaction of BV-2 microglia [201]. Water extract of *Eumoides ulmoides* bark ameliorates A*β*25-35-induced learning and memory dysfunction in mice and plays a neuroprotective role mediated by cholinergic protection and enhancement [202]. Lei et al. demonstrated an improved influence of PDG on A*β*1-42 neurotoxicity in mice. They found that PDG treatment remarkably inhibited the expression of TLR4 and NF-*κ*B upregulated by A*β*1-42, thereby suppressing the expression of TNF-*α* and IL-1*β*, and relieving inflammatory reactions, suggesting that PDG is involved in the regulation of the TLR4/NF-*κ*B pathway [203]. This finding highlights the neuroprotective activity of PDG, which decreases neuroinflammation by modulating the TLR4/NF-*κ*B pathway, thus reducing cognitive dysfunction in AD mice, suggesting that PDG is a potential drug for AD treatment.

*(3) Geniposidic acid (GPA)*. Geniposidic acid (GPA) is an iridoid glycoside isolated from *Eucommia ulmoides*. Several studies have shown that GPA exhibits anti-inflammatory, antioxidant, antiaging, and neurotrophic roles. Zhou et al. found that the distinct contribution of GPA to the APP/PS1 transgenic AD mouse model was partly regulated by its anti-inflammatory effect. They confirmed that GPA could ameliorate A*β* plaque load and learning and memory function in APP/PS1 mice by blocking the TLR4/2–MyD88 signaling pathway in reactive microglia when HMGB-1 protein expression was reduced [204]. This finding provides theoretical support for the application of GPA in the treatment of AD.

*(4) Curcumin*. Curcumin is the yellow component of the spice turmeric isolated from the root of turmeric. Studies have shown that curcumin has powerful anti-inflammatory, antioxidant, and antiamyloidosis activities and may play neuroprotective roles in AD [205–208]. Curcumin could reduce the expression levels of HMGB1, TLR4, or TLR2 in lipopolysaccharide-treated human endothelial cells [209]. He et al. found that curcumin pretreatment significantly inhibited the expression of TLR4 and RAGE in A*β*25-35-induced microglia. Curcumin also significantly reduced the expression of TNF-*α* and IL1*β*. These results suggest that curcumin may inhibit HMGB1-mediated inflammation in microglia stimulated by A*β*25-35, partly by suppressing the expression of TLR4 and RAGE [210]. In conclusion, TLR4 suppression is a critical target of curcumin in treating AD.

*(5) Chotosan (CTS)*. Chotosan (CTS) is a traditional compound herbal formula consisting of 11 raw herbs often used to improve chronic headaches and high blood pressure symptoms and treat related neurological diseases [211]. Various animal models showed that CTS could effectively alleviate cognitive dysfunction and memory deficits associated with disorders that involve more or less abnormal microglial activation and inflammation of the CNS, such as chronic cerebral hypoperfusion, ischaemic stroke, diabetes, and aging. Rhynchophylline and isorhynchophylline, derived from *Uncaria sinensis*, are representative herbal components of CTS. They had preservative effects against A*β*25–35-induced cellular neurotoxicity; moreover, they inhibited LPS-induced proinflammatory factors, such as TNF-*α* and IL-1*β* and NO produced by mouse N9 microglia [212]. Although clinical practice showed that CTS could treat cognitive impairment and behavioral and psychological symptoms in patients with AD, the specific pharmacological mechanisms needed to be clarified. Chen et al. found that CTS administration decreased the level of TLR4 induced by A*β*1–42 and inhibited TLR-4-mediated inflammatory reaction. The production of NF-*κ*B p65, TNF-*α*, and IL-1*β* was reduced in the hippocampus. A*β*1–42 enhanced the activity of caspase-3 and reduced the Bcl-2/Bax ratio, leading to obvious apoptotic reactions in the hippocampus. However, CTS treatment showed strong antiapoptotic effects by reversing the upregulation of proapoptotic proteins [213]. These results demonstrates that CTS therapy can ameliorate A*β*1–42-induced learning and memory disorder through neuroinflammation and apoptosis mediated by the TLR-4/NF-*κ*B signal pathway. This finding provides an idea for the treatment of CTS in AD.

#### 5.6.2. Electroacupuncture

Acupuncture originates from ancient China and is one of the oldest treatments worldwide. Acupuncture, a part of traditional Chinese medicine (TCM), has been an alternative and complementary therapy for over millennia. It belongs a complement of traditional medicine in various eastern and western countries recently [214]. Electroacupuncture (EA) combines acupuncture and electrical stimulation to treat multiple diseases effectively. Acupuncture therapy has a beneficial effect in mitigating cognitive impairment in AD [215, 216]. Recent research has shown that EA treatment improves neuroinflammation and thus cognitive dysfunction in mice with AD [217, 218]. EA therapy inhibited microglial activation, which showed that electroacupuncture at GV 20 and ST 36 points could relieve neuroinflammation effectively and thus reduce cognitive impairment. Previous research has also reported that EA at ST36 represses TLR4/NF-*κ*B signaling, thereby reducing LPS-regulated inflammation in rat models [219]. Moreover, acupuncture could regulate the gut microbiome [220]. He et al. demonstrated that inflammatory substances produced by dysbiosis of intestinal flora could trigger subsequent cerebral inflammatory reactions. They also observed that preventive EA therapy during ageing could rescue learning and memory dysfunction in ageing rats via modulating the gut microbiome and impaired microbiome-gut-brain axis. Furthermore, this protective effect of EA might be related to the downregulation of the TLR4/NF-*κ*B signaling pathway [221]. In conclusion, the neuroprotective effect of EA in suppressing the TLR4 pathway activation may be an effective treatment for AD.

## 6. Conclusion

Although therapeutic targeting of TLR4 in AD models has yielded promising results, further research is needed to translate these approaches into clinical practice. The design and optimization of agonists that selectively target the TLR4-MyD88 pathway may be a promising future agent that can preferentially enhance the phagocytic activity of glial cells while moderately upregulating glial cytokines and cytotoxins. Since the activation of TLR4 may modulate neuroinflammation and promote glial scarring, further preclinical and clinical safety research is needed before recommending these agents for clinical trials.

Moreover, the key adverse activities of TLR4 antagonists may be associated with their inhibition of abnormal protein and cell fragment clearance and interference with myelination. One challenge facing the clinical development of some TLR4-specific agonists and antagonists is their large molecular structure, which may prevent them from crossing the BBB. Besides, the effectiveness of therapeutic strategies targeting TLR4 may rely on the stage of the disease. For instance, prevention of A*β* accumulation with TLR4 agonists may be beneficial only in early AD. Therefore, early AD diagnosis is the key to effective treatment of TLR4. Thus, the specific involvement of TLR4 in disease staging in AD pathology is an important goal. The imbalance of the immune system in AD is complex, with interactions and interacting factors affecting neuroinflammation. Therefore, a single inflammatory mediator may not be entirely harmful or beneficial. In the future, it will be significant to explore the mechanisms between TLR4 and other receptors to determine effective therapeutic strategies for AD.

## Figures and Tables

**Table 1:** Summary of AD therapeutic approaches targeting TLR4.

Intervention	Animal model	Treatment	Mechanism	Reference
Hesperetin	A*β*1-42-induced AD model in C57BL/6N mice	50 mg/kg treatment for 6 weeks	(a) Inhibiting oxidative stress by reducing LPO and ROS and increasing Nrf2 and HO-1 (b) Inhibiting neuroinflammation by reducing TLR4, p-NF-*κ*B, TNF-*α*, and IL-1*β* (c) Inhibiting apoptosis by reducing Bax, caspase-3, and PARP-1 (d) Reducing memory dysfunction by increasing the levels of syntaxin, SNAP-25, PSD-95, Syp, and SNAP-23	[129]

Soybean isoflavone (SIF)	A*β*1-42-induced AD model in Wistar rats	80 mg/kg treatment for 14 days	(a) Improving learning and memory skills (b) Inhibiting levels of proinflammatory factors TNF-*α* and IL-1*β* (c) Inhibiting A*β*-induced elevation of TLR4 levels and NF-*κ*B expression in the nucleus	[131]

GX-50	APP transgenic AD model	1 mg/kg/day for 2 months at 5 months of age	(a) Inhibiting the expression of TNF-*α*, IL-1*β*, NO, PGE2, and iNOS and COX-2 in microglia of A*β*-treated rats (b) Inhibiting microglia activation and the expression of IL-1*β*, iNOS, and COX-2 in APP transgenic mice (c) Inhibiting the activation of NF-*κ*B and MAPK cascades (d) Reducing TLR4, MyD88, and TRAF6 expressions in vitro and in vivo	[73]

ProBiotic-4	9-month-old senescence-accelerated mouse prone 8 (SAMP8) mice	2 × 10^9^ CFU once daily for 12 weeks	(a) Reducing IL-6 and TNF-*α* levels, plasma, and brain LPS concentrations, TLR4 expression and NF-kB nuclear translocation in the brain, resulting in improved cognitive dysfunction in aged mice	[153]

MG136-pMG36e-Glucagon-like peptide-1 (GLP-1)	LPS (0.25 mg/kg) for 7 days in male C57BL/6 mice	Administered in drinking water for 14 days	(a) Attenuating neuroinflammation and improving LPS-induced memory impairment by downregulating the TLR4/NF-*κ*B pathway	[159]

TAK-242	Male APP/PS1 transgenic mice	2 mg/kg/day for 28 successive days	(a) Inhibiting TLR4 and Bax levels, significantly improves neurological function (b) Promoting the conversion of microglia from the M1 to the M2 phenotype (c) Inhibiting MyD88/NF-*κ*B and NLRP3 signaling pathways	[33]

Cattle encephalon glycoside and ignotin (CEGI)	Male APP/PS1 transgenic mice; C57BL/6J mice	6.6 ml/kg/day CEGI for 30 days	(a) Inhibiting TLR4 expression and NF-*κ*B p65 phosphorylation to exert antineuroinflammatory effects	[173]

Fasudil	Male APP/PS1 transgenic mice	25 mg/kg/day for 2 months	(a) Inhibiting microglia activation and promoting their conversion to an anti-inflammatory phenotype by suppressing TLR4, MyD88, and NF-*κ*B expression, and promoting astrocyte conversion from A1 to A2 phenotype	[179]

Atorvastatin	A*β*1-42-induced AD model in Sprague-Dawley male rats	5 and 10 mg/kg from 3 weeks before to 6 days after A*β*1–42 injections	(a) Reducing TLR4, TRAF6, and NF-*κ*B levels, inhibiting microglia and astrocyte activation, and improving spatial learning ability and memory impairment	[37]

Tetrandrine	APP/PS1 transgenic 5XFAD mice; A*β*1-42-induced BV2 cells	10, 20, and 40 mg/kg every 2 days from the age of 5 months to 7 months	(a) Dose-dependently improving cognitive performance in mice (b) Promoting reduced amyloid plaque deposition and hippocampal apoptosis in the brain (c) Inhibiting the expression of inflammation-related genes (TNF*α*, IL-1*β*, and IL-6) and TLR4, p65, iNOS, and COX-2	[193]

Pinoresinol diglucoside (PDG)	A*β*1-42-induced AD model in BALB/c mice	5 and 10 mg/kg every day for 3 weeks	(a) Reversing A*β*1-42-induced memory impairment in mice (b) Inhibiting the release of proinflammatory cytokines (TNF-*α* and IL-1*β*), ROS, and MDA and promotes the activity of antioxidant enzymes (SOD and CAT) (c) Upregulating the ratio of Bcl-2/Bax and downregulates the expression of Cyt C and cleaved caspase-3, thereby inhibiting neuronal apoptosis (d) Reducing TLR4 expression and NF-*κ*B p65 activation and promotes Nrf2 and HO-1 expression	[203]

Geniposidic acid (GPA)	APP/PS1 transgenic C57BL/6J mice	25, 50, and 75 mg/kg every day for 90 days	(a) Improving spatial learning and memory abilities and reducing brain A*β* deposition in mice (b) Inhibiting the activation of astrocytes and microglia, downregulating the expression of proinflammatory cytokines and iNOS, and upregulating the expression of anti-inflammatory cytokines and Arg-1 (c) Downregulating the expression of TLR2, TLR4, RAGE, MyD88, and NF-*κ*B p65	[204]

Chotosan (CTS)	A*β*1-42-induced AD model in ICR male mice	375, 750 mg/kg/day for 3 weeks	(a) Improving memory impairment in mice (b) Decreasing TLR-4 and NF-*κ*B p65 expression and reducing the release of proinflammatory cytokines (including TNF-*α* and IL-1*β*) in the hippocampus (c) Increasing the Bcl-2/Bax ratio and decreasing caspase-3 activity, thereby inhibiting neuronal apoptosis	[213]
